# Implementation of maternal and perinatal death surveillance and response (MPDSR) in humanitarian settings: insights and experiences of humanitarian health practitioners and global technical expert meeting attendees

**DOI:** 10.1186/s13031-022-00440-6

**Published:** 2022-05-07

**Authors:** Neal Russell, Hannah Tappis, Jean Paul Mwanga, Benjamin Black, Kusum Thapa, Endang Handzel, Elaine Scudder, Ribka Amsalu, Jyoti Reddi, Francesca Palestra, Allisyn C. Moran

**Affiliations:** 1 London, UK; 2 Jhpiego, Baltimore, MD USA; 3 Hôpital Générale de Mweso, Nord Kivu, Democratic Republic of the Congo; 4 Médecins Sans Frontières, Amsterdam, The Netherlands; 5 Centre for Disease Control and Prevention, Atlanta, GA USA; 6 International Rescue Committee, New York, NY USA; 7 University of California San Francisco, San Francisco, CA USA; 8 World Health Organization, Geneva, Switzerland

**Keywords:** Humanitarian, Maternal, Perinatal, Mortality, Surveillance, Review, Response

## Abstract

**Background:**

Maternal and perinatal death surveillance and response (MPDSR) is a system of identifying, analysing and learning lessons from such deaths in order to respond and prevent future deaths, and has been recommended by WHO and implemented in many low-and-middle income settings in recent years. However, there is limited documentation of experience with MPDSR in humanitarian settings. A meeting on MPDSR in humanitarian settings was convened by WHO, UNICEF, CDC and Save the Children, UNFPA and UNHCR on 17th–18th October 2019, informed by semi-structured interviews with a range of professionals, including expert attendees.

**Consultation findings:**

Interviewees revealed significant obstacles to full implementation of the MPDSR process in humanitarian settings. Many obstacles were familiar to low resource settings in general but were amplified in the context of a humanitarian crisis, such as overburdened services, disincentives to reporting, accountability gaps, a blame approach, and politicisation of mortality. Factors more unique to humanitarian contexts included concerns about health worker security and moral distress. There are varying levels of institutionalisation and implementation capacity for MPDSR within humanitarian organisations. It is suggested that if poorly implemented, particularly with a punitive or blame approach, MPDSR may be counterproductive. Nevertheless, successes in MPDSR were described whereby the process led to concrete actions to prevent deaths, and where death reviews have led to improved understanding of complex and rectifiable contextual factors leading to deaths in humanitarian settings.

**Conclusions:**

Despite the challenges, examples exist where the lessons learnt from MPDSR processes have led to improved access and quality of care in humanitarian contexts, including successful advocacy. An adapted approach is required to ensure feasibility, with varying implementation being possible in different phases of crises. There is a need for guidance on MPDSR in humanitarian contexts, and for greater documentation and learning from experiences.

**Supplementary Information:**

The online version contains supplementary material available at 10.1186/s13031-022-00440-6.

## Background

Humanitarian settings often represent situations of high risk for pregnant women and newborns [1–3], yet maternal deaths, stillbirths and neonatal deaths are challenging to measure or estimate in these settings [4]. Information on maternal deaths, stillbirths and neonatal mortality may be under-utilized in guiding and informing humanitarian responses, leading to missed opportunities for prevention.

Attempts to use population-based surveys to improve quantitative measurement of maternal deaths in humanitarian settings have been made, recently including methodologies such as Reproductive Age Mortality Surveys (RAMOS) [5–10], and ‘RAPID’ methodology to identify under-reported deaths in facilities [11, 12]. Whilst these have led to improvements in the data, significant under-reporting remains, and estimates of maternal mortality in humanitarian settings are often inaccurate or misleading [13]. Perinatal deaths have received less attention in mortality surveys in humanitarian settings; the focus of mortality reporting has generally been on crude or under 5 mortality [14], with a lack of reporting of stillbirths, and limited differentiation or identification of the neonatal category as a proportion of under 5 deaths [15]. The challenges in obtaining accurate quantitative information on maternal and perinatal deaths using survey methodologies highlight the importance of maximizing and utilizing both quantitative and qualitative information from routine data sources from both national ministries of health and humanitarian organizations.

**Fig. 1:**
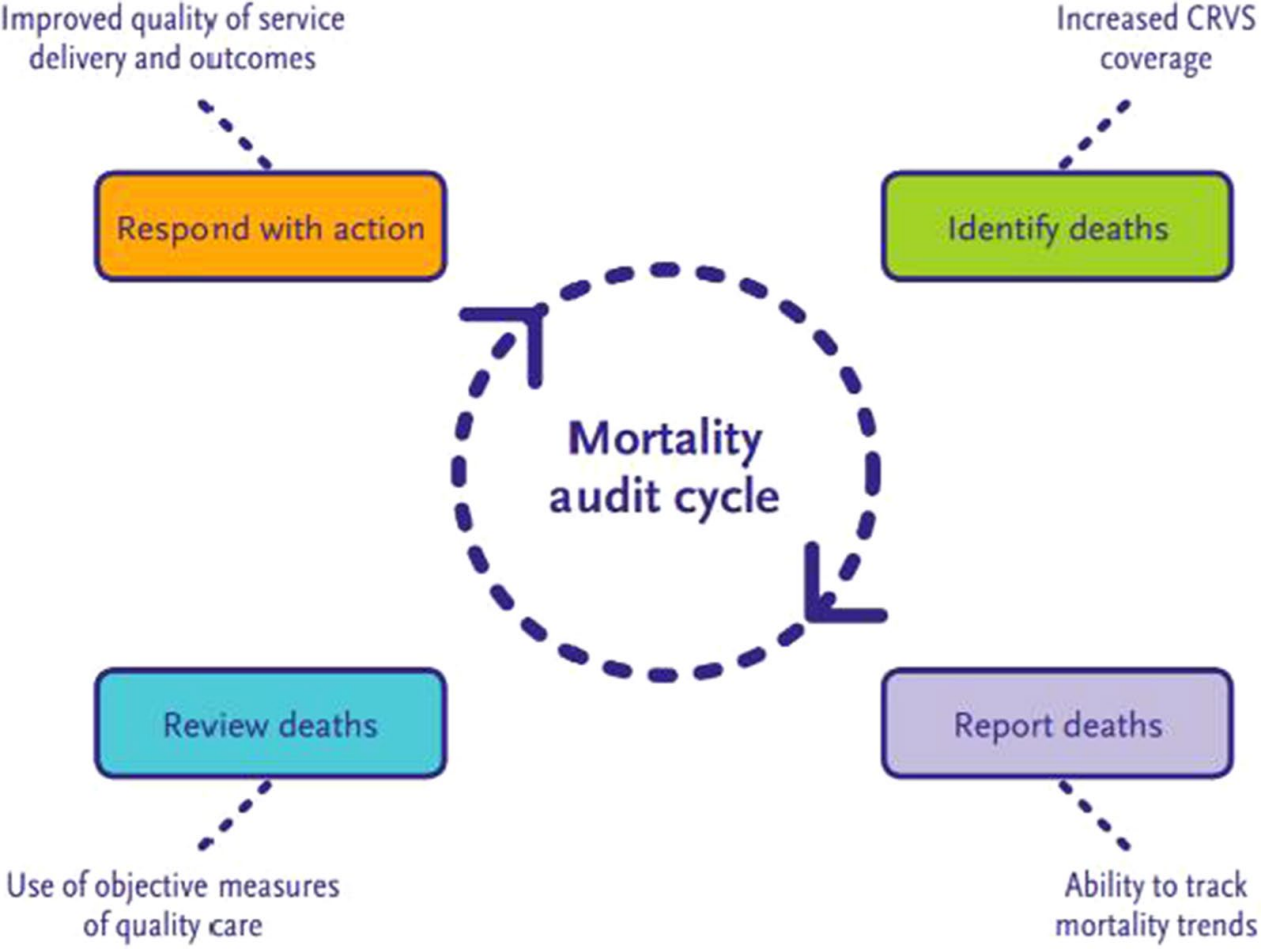
Mortality audit cycle

Maternal and perinatal death surveillance and response (MDPSR) is the system of identifying maternal and perinatal deaths, reporting them to relevant actors, learning lessons from qualitative and in-depth root-cause analysis of the causes and circumstances surrounding these deaths (mortality review), and responding with actions to prevent future preventable death (Fig. 1) [16]. The MPDSR approach has been recommended by the World Health Organization (WHO) and incorporated into technical guidance for maternal [16] and perinatal deaths [17] released in 2013 and 2016 respectively, with adoption in national policy in 126 and 100 countries respectively by 2018/2019 [18].

Despite the intuitive theoretical benefit of the system, the MPDSR approach is not an intervention whose efficacy can easily be proven in research studies [19]. There is evidence to suggest that maternal and perinatal death reviews, when implemented alongside training and development of local leadership might lead to significant reductions in maternal and perinatal mortality [20–22], however this is likely to be dependent on the quality of the process being undertaken. In low resource settings where the numbers of maternal and perinatal deaths are the highest, significant challenges have been identified in the MPDSR process, including the quality of mortality reviews and linkage to concrete actions [23, 24]. It has been argued that in settings with high mortality rates and limited resources the main causes of death in women and newborns are already understood, and providing direct clinical care should be prioritized over investment in review mechanisms [25]. This same argument could be applied to humanitarian settings where competing priorities are significant, and the need for immediate action is great. At the same time, a counter-argument could be made that ensuring fit-for-purpose mechanisms for reporting deaths are in place from the earliest days of an emergency, and that using available data for continuous improvement of health services are core components of humanitarian response. Deeper understanding of experiences implementing MPDSR in diverse humanitarian settings is needed to inform conclusions.

As a first step in addressing the paucity of literature on MPDSR implementation in humanitarian settings, this paper documents insights and experiences of maternal and newborn health and humanitarian response practitioners consulted in preparation for a global expert meeting to explore MPDSR implementation experiences in a range of different humanitarian settings including those with armed conflict, refugee and/or internally displaced populations, recent or recurrent natural disasters, and infectious disease epidemics, with varying levels of pre-crisis national implementation of an MPDSR system.

## Expert consultation

### Consultation methods

A two-day expert consultation was held in New York City on 17–18 October 2019 with 49 participants from academia, government, UN agencies and non-governmental organizations to review lessons learned from past and ongoing initiatives and collectively agree on if and how to advance MPDSR implementation in humanitarian settings.

To inform discussions at the consultative meeting, a series of semi-structured in-depth interviews were conducted with 55 purposively selected individuals meeting at least one of two criteria: (1) clinical, academic or programmatic experience in maternal and/or newborn health in humanitarian settings, and/or (2) programmatic or research experience in MPDSR. An initial list of interviewees and countries of focus was identified by a steering committee responsible for planning the expert consultation, and this was followed by a snowballing sampling [26] approach to identify further interviewees based on the recommendations of previously identified contacts. The majority of interviewees were from non-governmental organisations [27], followed by academic institutions [12], united nations [6], and ministries of health [4]. All interviews were conducted by the first author who was a paediatrician with humanitarian experience with NGOs, working as an independent consultant. Interviews were conducted in person, via video conference or by telephone using a semi-structured interview guide developed specifically for this purpose, in consultation with the aforementioned steering committee (Appendix 1). Four interviews included groups of 2–4 people and the remaining interviews were with individuals. Participants gave verbal consent after an explanation of the purpose of the interview, could discontinue interviews at any time if requested, and were given the option to remain anonymous. Interviews were between 30 min to one hour and were not audio-recorded; insights and experiences shared by participants were captured in interview notes taken by the first author/interviewer.

In order to supplement and contextualize respondent descriptions, identify additional studies and reports of MPDSR in humanitarian settings, and triangulate information, interview participants were asked to share any previous unidentified published or unpublished reports on MPDSR implementation in humanitarian settings. Documents reviewed alongside interview findings are presented in Tables 1, 2, 3. In addition, reference lists of an ongoing Cochrane systematic review [22] were searched to identify any studies including humanitarian settings, and interviewees were asked to recommend additional studies or grey literature.

Interview notes and documents were read and re-read to identify themes, and were organized by setting by the interviewer in order to develop case examples. Interview findings were then triangulated against the grey literature, and thematic analysis was undertaken to explore similarities and differences in challenges and lessons learned across diverse humanitarian settings.

At the two-day meeting, a summary of challenges and lessons learned shared by interview participants was presented and discussed. Through plenary discussions, panel discussions and group work, meeting participants discussed rationales for MPDSR implementation in acute and protracted humanitarian settings as well as in refugee camps or settings with displaced populations on the move and agreed on broad recommendations for future program planning, research and guidelines development.

Case examples used to illustrate experiences in MPDSR implementation in humanitarian settings are presented in Table 1, and common challenges identified across settings in Table 2. Although we present common themes by type of humanitarian context, it is important to note that some challenges shared may be country or program specific and not generalizable to other settings with similar humanitarian contexts; findings presented should be read as illustrative examples shared by interview and meeting participants. A more detailed summary of specific challenges and lessons learned identified in each setting is available in Appendix 3. In the sections that follow, we discuss key findings and implications for health program planning across diverse humanitarian settings.

**Table 1:** Case examples shared to inform discussion at expert consultation meeting

Country, year	Humanitarian context	National MPDSR policy status (WHO SRMNCAH Policy Survey, 2018–2019)	MPDSR implementation status (described by key informants)	Topics addressed	Data sources
Afghanistan, 2018–2019	Protracted conflict, internal displacement	National policy/law on maternal and neonatal death reviews in place MoH reports having subnational panels to review maternal deaths and facility-level process to review neonatal deaths	Limited implementation for maternal and neonatal deaths	Maternal and neonatal death reporting, maternal and neonatal death reviews	Interviews, Document review [28]
Bangladesh refugee camps, 2017–2019	Refugee camp	National policy/law on maternal and neonatal death reviews in place MoH reports having national and subnational panels to review maternal deaths and facility-level process to review neonatal deaths	Established for maternal deaths in national system, limited implementation for maternal and perinatal deaths in refugee camp setting	Maternal and neonatal death reporting, maternal and neonatal death reviews	Interviews Document reviews [29, 30] including unpublished reports
Cameroon, Chad and Niger refugee camps, 2018–2019	Refugee camp	National policy/law on maternal death reviews in place in Cameroon, Chad, and Niger. Cameroon policy also in place for neonatal death and stillbirth reviews Cameroon and Chad MoH report having national and subnational panels to review maternal deaths. Cameroon also reports having facility-level process to review neonatal deaths and stillbirths	Limited implementation for maternal deaths	Maternal death reviews	Interviews, Document review [27, 31–33] including unpublished reports
Central African Republic, 2014–2019	Protracted conflict, internal displacement	*Not reported*	Very limited implementation for maternal deaths	Community-based mortality surveillance, abortion-related death reporting	Interviews, document review [34, 35] including unpublished reports
Democratic Republic of Congo (North Kivu & South Kivu provinces), 2009–2019	Protracted conflict, internal displacement	National policy/law on maternal and neonatal death and stillbirth reviews in place MoH reports having national panel to review maternal deaths	Limited implementation for maternal deaths	Maternal death reporting, maternal death reviews	Interviews, document review [36] including unpublished reports
Iraq, 2018–2019	Post conflict/protracted conflict, internal displacement	National policy/law on maternal and neonatal death reviews and stillbirths in place MoH reports having national and subnational panels to review maternal deaths and facility-level process to review neonatal deaths and stillbirths	Established national MDSR system Nationally led perinatal death pilot in stable districts since 2018, scale-up in 2019	Maternal and perinatal death reporting, maternal and perinatal death reviews	Interviews, document review [37–39] including unpublished reports
Jordan refugee camps, 2016–2019	Refugee camp	National policy/law on maternal death reviews in place MoH reports having national and subnational panels to review maternal deaths	Neonatal mortality reviews commissioned by UNHCR since 2016	Perinatal mortality reviews	Interviews, Document review [40] including unpublished reports
Kenya refugee camps, 2007–2010	Refugee camp	National policy/law on maternal and neonatal death reviews and stillbirths in place MoH reports having national and subnational panels to review maternal deaths and facility-level process to review neonatal deaths and stillbirths	Maternal death review system established in camp by UNHCR	Maternal death review	Interviews, Document review [41]
Mozambique, 2019	Post-natural disaster (cyclone)	National policy/law on maternal and neonatal death reviews in place MoH reports having national and subnational panels to review maternal deaths and facility-level process to review neonatal deaths	Limited national implementation for maternal deaths	Maternal death reporting & review	Interviews
Nigeria (Zamfara & Yobe States), 2013–2018	Acute conflict, internal displacement	National policy/law on maternal and neonatal death and stillbirth reviews in place MoH reports having national and subnational panels to review maternal deaths and facility-level process to review neonatal deaths and stillbirths	Limited implementation for maternal and perinatal deaths		Interviews, document review [42, 43]
Sierra Leone, 2014–2019	Ebola/post-Ebola	National policy/law on maternal death reviews in place MoH reports having national and subnational panels to review maternal deaths	Developing national MPDSR system post-Ebola	Maternal and perinatal death reporting, maternal and perinatal death reviews	Interviews, document review [44–46]
South Sudan, 2013–2018	Acute & protracted conflict, internal displacement	National policy/law on maternal death reviews in place MoH reports having subnational panels to review maternal deaths and facility-level process to review neonatal deaths	Very limited national implementation for maternal deaths	Community-based surveillance, facility-based maternal death reviews	Interviews including unpublished reports
Syria, 2015–2017	Acute conflict	National policy/law on maternal and neonatal death reviews in place MoH reports having subnational panels to review maternal deaths and facility-level process to review neonatal deaths	Very limited implementation for maternal deaths	Maternal death review	Interviews

**Table 2:** Common challenges faced in MPDSR implementation identified across humanitarian settings

Humanitarian context	Identification and Reporting	Review and Response
Acute crises	Reporting anecdotal during acute disaster response Decline in reporting after onset of insecurity Reported mortality rates likely to be large under-estimates, and paradoxically reduce while true mortality rates rise Remote reporting via SMS may be more resilient Often very limited reporting of community deaths Low skilled birth attendance rates early after displacement	Very challenging to conduct formal death reviews during acute response period Death reviews were feasible in IDP camps a few months after acute conflict/displacement External facilitation (mentorship visits) to support reviews was useful but often interrupted by insecurity Experienced staff trained to conduct reviews often leave region and system may collapse after insecurity Indirectly/informally highlighting learning points from recent deaths during trainings may be more feasible than conducting formal mortality reviews
Protracted crises	Insecurity and access limit community reporting Stillbirth and neonatal death misclassification common in community death reporting Community surveillance often does not include maternal or perinatal deaths Simplified definitions in community surveillance may miss indirect, early pregnancy and postpartum deaths in particular Deaths ‘in transit’ between facilities often not reported by either referring or receiving facility	Accurate cause of death determination for maternal and perinatal deaths identified via community-surveillance was challenging Verbal autopsy may not be possible or prioritized Facility-based review of maternal deaths revealed unexpectedly high proportion of deaths due to unsafe abortions Defensive approach, reviews evolving into HR processes, linked to disciplinary procedures Even where maternal death review well established (with no-blame culture), perinatal death reviews have had limited implementation, and limited engagement of some staff and challenges with ICD-PM coding Security of health workers may be at risk if blamed for deaths, particularly when confidentiality and anonymity is challenging Community tensions may increase sensitivity of death reviews
Disease epidemics	Significant underreporting of maternal deaths Those with potential symptoms of the epidemic disease prioritized above other causes Non-epidemic health issues often deprioritized Mistrust and suspicion of health services reduces reporting Linking maternal death reporting with integrated disease surveillance and response (IDSR) may improve reporting Short term funding during epidemics may improve mortality surveillance, but this may not be sustained without predictable investment in health system strengthening	As in other crises formal death reviews impacted by crisis Focus on disease epidemic means reviewing other causes of death requires explicit focus and investment Funding should continue to support reporting and review of non-epidemic related deaths
Refugee camps or camp-like settings	Fear of loss of household rations if death reported Deaths often not captured if woman referred outside refugee camp for care Refugee mortality often underestimated, and national statistics may exclude or not differentiate refugee deaths Accountability and focus on improving care within camp may be reduced if mortality statistics are exported outside the camp	Lack of experienced staff with capacity and authority to do mortality reviews (particularly with prescriptive guidelines on composition of review committees) High staff turnover Deaths often occur outside camps in higher facilities often not reviewed by health services within camps, and lessons learned often not fed back to referring facilities
Any setting	Mortality statistics unlikely to represent true mortality rates (underreporting of facility and community deaths) Concerns about negative consequences discourages reporting Concerns about reputational damage if facility deaths reported Data collection may raise suspicion; medical records destroyed and false names used due to fear of data use by military/government actors Poor engagement/punitive approach towards TBAs discourages reporting of community deaths	Feasibility of review limited by data available and capacity/interests of staff involved Blame culture limits quality of reviews Limited attention to or documentation of response and follow-up Large numbers of perinatal death reviews difficult to manage Large committees with prolonged meetings are resource intensive, particularly in areas with strained human resources Sustainability of reviews without external donor support is questionable in some settings Limitations of clinical documentation limit value of reviews Reviews conducted by an external agencies may miss opportunity for local teams to be involved in defining solutions Some recommendations useful, others had tendency to be generic and non-specific

Challenges are those discussed in case examples; some may be context specific and not generalizable to country-wide or to other countries with similar humanitarian contexts

### Key findings: death identification and reporting

Most challenges identified related to identification and reporting of maternal and perinatal deaths are not unique to humanitarian settings, but may be more pervasive or exacerbated by political instability, livelihood uncertainty, and health system constraints in crisis-affected settings.

#### Disincentives to reporting

A common theme in interviews was the suggestion that in health facilities, reporting poor outcomes such as maternal and perinatal deaths may be disincentivized due to concerns about scrutiny and reputational damage for individuals, organizations and governments. This was mentioned by interviewees working across all sectors including ministry of health officials, but interviewees also highlighted the situation where organizations could fear that reporting deaths may reflect badly upon them and lead to loss of contracts with donor organizations. This disincentive may not often be balanced by significant incentives to report deaths, as accurate reporting of deaths is not always followed by increased support, or explicitly valued by donor organizations or communicated as a priority. The concept of accurately reporting deaths being a marker of good quality care is perceived as difficult to communicate at all levels, especially given that improved reporting may lead to increasing death rates, reflecting poorly on individuals and organizations.

At the community level, death reporting is often further limited, or may be non-existent, leading to significant underestimation of deaths. It may also be disincentivized due to fear of reputational damage amongst community leaders, and social hierarchies may discourage reporting of deaths. Several interviewees suggested that mistrust between formal health providers and traditional birth attendants (TBAs) may be particularly detrimental to efforts to report community deaths, but beyond this, general mistrust in health services, as has been seen in recent Ebola epidemics, may also discourage reporting. In some cases, punitive policies towards TBAs or policies such as fines for home births may further decrease reporting of community deaths.

In refugee camps, interviewees pointed out that reported maternal and neonatal mortality rates have often been shown to be lower than in host populations, and this has been demonstrated in the literature [27]. However, although surveillance and population estimation may often be more feasible in camp settings, several factors were raised by interviewees which may lead to underestimation of mortality. Fear of loss of assistance after reporting a death is a particular concern, and inflated population estimates may lead to underestimates of maternal and perinatal mortality. Indeed maternal death reporting has been variable, and neonatal mortality in particular has been shown to be significantly underestimated by camp surveillance [31].

If an MPDSR system is conducted without strong leadership and support in place to explain the purpose of the system and ensure positive engagement with health-workers and communities and avoidance of a blame approach, MPDSR efforts may have the paradoxical effect of reducing reporting of deaths. Interviewees cited examples of practices to avoid reporting of deaths such as falsification or hiding of medical records, and in some cases potentially unfeasible reductions in deaths were seen after MPDSR implementation. Reliance on paper-based systems was suggested as a factor allowing deaths to be more easily concealed.

#### Deaths after referral, or in ‘transit’

A common theme identified in interviews was under-reporting of deaths occurring ‘in transit’, or misclassification of deaths to avoid reporting among facility statistics. In particular, late referrals to higher facilities before death were raised by several interviewees. Indeed, many facilities providing basic emergency obstetric care (BEmONC) in humanitarian settings report few deaths of pregnant women in particular, as they are likely to be referred before death to a higher facility. In many cases, if the woman dies after referral, her death and/or that of her baby is often not reported in the BEmONC facility, and may not contribute to mortality statistics for her population, particularly if referral occurs from within a camp to a higher facility outside, leading to under-reporting.

In referral facilities, early deaths after admission are also known to often be reclassified as transit deaths, and this is sometimes even reinforced with arbitrary policies such as systematically classifying any death within 24 h of admission to be a transit death, with blame shifted to lower facilities which made the referral. Extreme examples were also given from interviewees of refusal of admission in referral facilities for women appearing likely to die, apparently with the motivation of avoiding damaging mortality statistics.

The overall effect of such practices is not only to underestimate mortality rates, but also to miss the opportunity for learning from deaths. This is particularly important in humanitarian settings where referral pathways are often weak or face unnecessary barriers, but may be allowed to continue to remain so without learning processes in place, leading to further unnecessary deaths.

#### Quality of data

Even when deaths are regularly reported, clinical documentation and mortality reporting frameworks often limit meaningful analysis, particularly for perinatal deaths which usually receive a lower priority than other deaths in humanitarian settings. The ‘minimum perinatal dataset’ as described in WHO guidance [17] is often unavailable or fragmented, resulting in missed opportunities to identify areas for improvement.

Clinical documentation in neonates is often particularly limited, with inaccurate or unreliable reporting of core indicators, and absent or ambiguous cause of death data. Aggregate neonatal mortality statistics are also often problematic. Mortality rates are often undifferentiated by weight categories, which is particularly important for analyzing comparisons, trends and quality of care in facilities given the marked differences in expected mortality [47]. This is also especially important given the variation in admission criteria for neonates in different humanitarian settings [48], as rationing admissions based on weight criteria has a significant impact on mortality statistics.

#### Under-reporting and misclassification of stillbirths

A theme throughout interviews was a lack of, or highly variable practices in reporting of stillbirths, with inconsistent differentiation into intrapartum and antepartum stillbirths. Misclassification of stillbirths as neonatal deaths and visa-versa, particularly in community surveillance methods, was also frequently noted. Nevertheless, examples did exist of stillbirth reporting, including examples of published research on stillbirth reporting in humanitarian settings [49].

Interpretation of stillbirth data was however identified as a challenge, with a lack of clarity on definitions of target stillbirth rates in humanitarian settings at facility level. This was identified as particularly problematic in settings with very low background caesarean section rates, where caesarean sections may be provided primarily for maternal indications and less commonly for fetal indications.

#### Under-reporting and misclassification of maternal deaths

Abortion-related and early pregnancy deaths were recognized as almost universally under-reported or misclassified in humanitarian settings, particularly in settings with legal restrictions on abortion. Unsafe abortion related deaths in facilities may be mis-coded as sepsis, or peritonitis for example, and women may be admitted in different areas of facilities, meaning mortality statistics from obstetric wards will miss these deaths. Community surveillance systems in crises generally focus on communicable diseases, and are less likely to report maternal or perinatal deaths [50]. When these systems include maternal mortality, they often miss early pregnancy deaths by their design, because simplified systems of reporting often only report peripartum deaths as maternal deaths. Relying on pregnancy surveillance, while rare in humanitarian settings, is also prone to missing early pregnancy deaths as women in these settings are likely to present late to antenatal care. A notable example from Central African Republic where a facility-based review identified 30% of maternal deaths as related to unsafe abortion, demonstrated that reporting these deaths can be linked to advocacy, awareness and political engagement in a legally restrictive and challenging ‘humanitarian’ setting [34].

Similar to the challenges in reporting early pregnancy deaths, indirect maternal deaths are often mis-reported as non-maternal deaths, either due to the design of data collection methods (in particular community-based surveillance reporting), or due to a lack of awareness or training of health workers, or indeed because non-maternal deaths may attract less scrutiny.

#### Politics of mortality data

Importantly, the question of who ‘owns’ population and health service data, how it can be shared, and indeed whether it can be reported publicly may be affected by the political context in humanitarian crises [51]. Maternal mortality in particular is often highly political, and interviewees noted that the publication of unfavorable mortality statistics is sometimes discouraged or prevented. Indeed mortality among refugee or displaced communities is often also omitted from national reporting, or not differentiated within overall statistics. Deaths among women who’s access to healthcare may have been affected by exclusionary policies (e.g. migrant women) may also be particularly politically sensitive.

### Key findings: review processes

Humanitarian contexts are often characterized by higher mortality rates, poorly functioning health systems, lack of resources and trained health workers, and extreme political, economic and social changes. Therefore, efforts to develop formal death reviews have been known to be interrupted after the onset of insecurity, when health systems collapse and trained health workers may leave an area. Interviewees reported that health workers who do remain may be overwhelmed with treating the living, and less priority may be placed on death reviews. Quality and leadership of deaths reviews may also be compromised by lack trained or experienced staff. In this situation, highly prescriptive national recommendations on the composition of large death review committees and extensive requirement for documentation may also present a barrier to conducting any reviews when the guidance is over-interpreted and the relevant staff are unavailable to conduct the reviews. Furthermore, in the context of challenging situations with high death rates, potential psychological and moral distress, reviews which consistently identify recommendations which the healthcare providers feel powerless to implement may be demoralizing, and a process with even a suggestion of blame may be particularly unwelcome and unhelpful in a context of high levels of stress.

#### Simplified verbal and social autopsy for community deaths

Simplified verbal autopsies linked to community surveillance system were described by interviewees. In several settings, standard tools were perceived as complex, and local adaptations were made. However these adaptations are largely pragmatic rather than evidence-based, and lead to under-reporting, underdiagnosis and misclassification, particularly of stillbirths, early neonatal deaths, and early pregnancy and abortion-related deaths. In this case a clear balance is needed depending on the level of training of the community health workers, as examples were also given where more complex tools led to less data collection (or none) due to the limited feasibility of the tool. Despite the limitations, verbal autopsy was described by interviewees as useful when feasible, despite the imperfect information which is obtained. Social autopsy was also perceived as useful particularly in settings where community and cultural factors limiting skilled birth attendance were important. These findings are also documented in a separate review of verbal and social autopsies in humanitarian settings [52].

#### ‘Blame culture’ in death reviews

MPDSR functions best in settings with a culture of accountability, learning and improvement. A culture of trust is nurtured by strong leadership and continuous re-assurance of a “blame-free culture” [24]. In several humanitarian settings, it appears that weak or partial implementation of MPDSR has left the system vulnerable to a blame approach being adopted, with the success of reviews highly dependent on local personalities and leadership. In humanitarian settings where trained staff may be lacking, the MPDSR system can perhaps be more easily misinterpreted as a tool for disciplining staff. Examples were given from both ministries of health and from within humanitarian organizations where due to lack of training or awareness amongst staff, the boundaries between the death review process and human resource management have become blurred in some cases, with information from reviews being linked to disciplinary procedures. Interviewees cited examples of how this approach has led to defensive and poor-quality reviews with staff reluctant to openly discuss the reasons for deaths, and limited useful recommendations as a result.

Security of staff is also a particularly important consideration in settings with a breakdown in normal legal protections for health workers. Health workers may face considerable risks of violence in humanitarian settings, and occasionally murder of healthcare workers has occurred due to perceived poor quality of care [53]. Community tensions may also contribute if one group is felt to have been treated differently by staff. Death reviews, if done well and without blame may not increase this risk, but the risk of inflaming tensions if conducted poorly should also be considered. The consultations and literature review did not identify sufficient examples of community engagement in death review committees in such conflict settings in order to provide recommendations, and this is clearly an area in need of further study.

Interviews suggested that the level of ‘institutionalization’ and awareness of MPDSR and its principles is highly variable between different organizations and individuals working in humanitarian settings in both governmental and non-governmental organizations. The interpretation of MPDSR as a tool in human resource management was mentioned as a common misunderstanding, leading to counter-productive approaches.

#### Limited focus on perinatal death reviews

Relatively few examples of formalized or regular in-depth perinatal death reviews in humanitarian settings could be identified from interviews, and stillbirths were identified as particularly neglected in examples from most interviewees. Perinatal death reviews are currently being rolled out in a number of the settings, but experience is limited. The concept that maternal death reviews are a priority to be established before perinatal death reviews was generally accepted. Although some examples exist of valuable reviews and recommendations, many interviewees cited concerns about the feasibility and sustainability of reviewing large numbers of perinatal deaths. Reservations from health-workers were also mentioned regarding the quality of perinatal death reviews, with discussions often remaining superficial, and with some lessons learnt having a tendency to be generic and predictable. The limited written clinical documentation of care for newborns was also mentioned as a factor limiting quality reviews. Many interviewees believed that in most settings with large numbers of deaths a system for selecting subsets of stillbirths and neonatal deaths for review would be required, alongside facility audits and identification of priority quality improvement themes. Suggestions were made that greater capacity building and familiarity with conducting perinatal death reviews is required to increase their quality and the value of the recommendations. Ultimately, a greater priority placed on preventing stillbirths and neonatal deaths would be required in the humanitarian sector in general.

A concern highlighted in some settings, particularly in protracted crises with unpredictable access to obstetric care, was that a focus on reviewing perinatal deaths may drive increases in caesarean section rates, which may be appropriate, but also may potentially increase the risk to women in future pregnancies if skilled birth attendance becomes unavailable. This may be particularly problematic in the context of potentially unreliable diagnosis of fetal distress. Ethical concerns were also highlighted, as in some humanitarian settings caesarean sections may be performed upon maternal indications alone rather than fetal indications, with perinatal deaths (and their review) being deprioritized in these contexts.

### Key findings: response to prevent future deaths

Interviewees highlighted that response to maternal and perinatal deaths depended entirely on the quality of the review process and the capacity and resources to implement recommendations. Poor quality reviews would often lead to generic and non-specific recommendations lacking clear actions or follow up., and lack of capacity or resources to implement recommendations was recognized as demotivating, and also negatively affected MPDSR processes.

Despite the challenges, interviews revealed several examples of quality death review processes with good leadership resulting in practical and positive recommendations and improvements in care, demonstrating the value of the approach even in extremely challenging circumstances [41]. Many of the findings and recommendations of reviews highlighted were common to more stable settings, such as improved documentation, partograph interpretation, blood bank strengthening, antibiotic prophylaxis for caesarean sections, and sensitization of women, communities and traditional birth attendants. Other examples were more specific to the humanitarian context and may be less well recognized in relation to MPDSR. Examples from interviewees are listed in Table 3.

**Table 3:** Examples of recommendations emerging from maternal and perinatal death reviews of particular relevance in humanitarian settings, mentioned by interviewees

Recommendations to address delays in decisions to seek care	Recommendations to address delays in reaching care	Recommendations to address delays in receiving quality care
Importance of community engagement, including in design of services + feedback Addressing marginalisation with representation of community members within services Culturally appropriate and respectful services paramount Communication with women regarding risk planning for unpredictable insecurity Information sharing with community on security situation and availability of safe transport routes Addressing misinformation regarding health service Improving public perception of humanitarian actors Improving trust in health facilities and staff, and communication of humanitarian principles of neutrality Ensuring right to healthcare access regardless of legal status De-linking of immigration enforcement from health care activity, including data separation	Addressing restrictions on movement due to military/camp security procedures for women in labor Negotiation of referral pathways dependent on negotiation with armed actors/military/security/camp management Birth planning in insecure settings (e.g. availability of maternity waiting homes in situations of unpredictable security) Availability of free/subsidised transport networks with actors with access to ‘humanitarian space’ Coordination and communication between health actors, and with non-health actors Decision-making on strategic placement of basic and comprehensive emergency obstetric and newborn care services to avoid exacerbating referral delays in times of insecurity	Recruitment and retention of adequate staff in insecure settings Remote support for improving quality of care Address cultural barriers to emergency interventions—e.g. advanced consent for caesarean section from women/family decision makers Blood bank strengthening and emergency community blood drive activities Strengthening triage procedures Strengthening coordination between actors Ensuring respectful maternal and newborn care

Despite some good examples, and despite the need for rapid implementation of recommendations in humanitarian contexts, a consistent theme was lack of systems for follow up of recommendations made during mortality reviews. Challenges for follow up in humanitarian settings include high turnover of staff, changing nature of crises and priorities, and short funding and planning cycles.

### Strengths and limitations of consultation

This consultation has important limitations, and was conducted as a first step in addressing gaps in documentation of MPDSR implementation in humanitarian settings. Although a wide participation was sought from individuals from diverse settings, interviewees were predominantly from the non-governmental sector, with fewer interviewees from ministries of health. In some case studies, limited numbers of interviewees could be identified from a specific setting, and a different balance of interviewees could be identified from each setting. Importantly, case examples should not be interpreted as representing the entire country from which they were derived. In addition, the background of the interviewer may inevitably have been associated with bias which is difficult to quantify or mitigate against, as their experience could also have influenced discussions which were not recorded. This findings from this consultation should be an impetus for further research rather than interpreted as a conclusive picture of MPDSR in humanitarian settings.

## Conclusions

Learning lessons from deaths in order to prevent them in the future has a clear justification and potential benefit. Indeed, neglecting to make an attempt to learn lessons from avoidable deaths serves to undervalue the lives that have been lost, and represents a significant missed opportunity [1, 3]. There are several characteristics of humanitarian situations which make a quality MPDSR process challenging at every step in the cycle, yet the case examples shared in this consultation suggest that the principles of MPDSR are relevant and useful in even the most challenging humanitarian settings. Nevertheless, a more flexible interpretation of the MPDSR approach may be required in humanitarian settings depending on the context and the phase of a crisis.

Strengthening of MPDSR in humanitarian settings requires an explicit focus, with support, leadership and good communication. Policies which may inadvertently disincentivize community level reporting should be avoided, such as linkage of death reporting to rations in refugee settings, or criminalization of traditional birth attendants. At all levels, trust between communities and health actors, as well as trust within systems is required to facilitate reporting, and the purpose of reporting should be clearly understood and evidenced in practice, with positive and supportive responses to reporting. Overall, greater research into the role of communities and community health workers in MPDSR processes would be beneficial.

Importantly, maternal and neonatal mortality rates reported from surveillance in humanitarian settings should be interpreted with caution, as many factors may limit their accuracy, and low mortality rates or declining trends from surveillance data may be falsely reassuring. Training and capacity building in reporting of early pregnancy, indirect maternal deaths and stillbirths, and simplified use of mortality groupings in systems such as ICD-PM and ICD-MM is required, and interpretation of mortality statistics should recognize the systematic weaknesses in these specific areas. In general, improved reporting of maternal and perinatal deaths would also need to be accompanied by a greater focus on monitoring quality of care and health outcomes in the humanitarian sector, as the focus is often on reporting activity inputs and coverage [54].

Flexible approaches may be required, for example with smaller death review committees when standard national recommendations on membership quota cannot be fulfilled due to lack of human resources, or combining teaching and mortality review in hybrid approaches, provided that leadership is effective and supportive in all cases. In conflict settings in particular, mortality review processes require consideration of their potential impact on the security of health-workers. Mentoring and support of death reviews and committees may also need to be more resilient to insecurity and access issues, and utilize remote communication or virtual platforms.

Limited capacity may mean the priority is to review all maternal deaths, and to report all stillbirths and neonatal deaths, but to review only a subset of perinatal deaths after selection of cases either randomly or selectively, or by themes identified during retrospective audits and case note reviews by key persons.

Where possible, efforts should be in partnership with host governments, supporting national MPDSR systems and using common reporting methods, while also explicitly including displaced and refugee populations. However non-governmental organizations should also strengthen their own MPDSR processes and use learning from these to inform better humanitarian responses. In particular, systems for documenting, following up and rapidly implementing recommendations from mortality reviews should become standardized and institutionalized and linked to accountability, without replacing or weakening national systems where these can be reinforced. Mechanisms for sharing of lessons learned both vertically within organizations and horizontally across different settings and organisations may have the potential to improve quality of care and inform humanitarian responses for women and newborns in general, including advocacy.

It is clear that MPDSR is potentially a valuable approach in humanitarian settings. A health systems approach could be used, recognizing that different aspects of MPDSR are of varying feasibility depending on the phases of crises and the extent of underlying national health systems and MPDSR implementation. Based on these interviews and discussions at the expert consultation, the below recommendations were agreed:

1. In acute humanitarian settings, a full MPDSR process may not be feasible or a priority. Tracking deaths is crucial, and the focus should be on counting deaths and establishing health services.
2. In protracted humanitarian settings, MPDSR could be implemented assuming that health services are in place. It is essential to ensure equal energy for establishing death surveillance as well as quality death reviews and implementing recommendations to improve the quality of services. The focus should first be on establishing the MPDSR system in health facilities, then move to surveillance and response of community-based deaths.

Importantly, this work highlighted the extremely limited documentation of experiences in MPDSR in humanitarian contexts, and the extent to which it’s role in these contexts is yet to be well defined. Further piloting and implementation research is required to explore feasibility of MPDSR in different humanitarian contexts to inform effective, flexible and context-appropriate approaches. Ultimately, greater accountability, and increased investment in quality of care across the humanitarian sector is required, in addition to greater attention to maternal and perinatal deaths. MPDSR could be one of the mechanisms for directing that focus in certain contexts, and for preventing future avoidable deaths (Additional file 1).

## Supplementary Information

**Additional file 1.** Semi-structured key informant interview guide.

## Data Availability

Data generated and analyzed during this study is included in the published article and its supplementary files. Additional details are available from the corresponding author on reasonable request.

## Abbreviations

MPDSR: Maternal and perinatal deaths surveillance and response
NGO: Non-governmental organization
RAMOS: Reproductive Age Mortality Study
TBA: Traditional birth attendant
