# Supplementary material for: Implementation of maternal and perinatal death surveillance and response (MPDSR) in humanitarian settings: insights and experiences of humanitarian health practitioners and global technical expert meeting attendees
Source: Confl Health. 2022 May 7;16:23. doi: 10.1186/s13031-022-00440-6 (PMC9077967; doi:10.1186/s13031-022-00440-6)
Supplement: Supplementary file 1 — Additional file 1. Semi-structured key informant interview guide. [file 13031_2022_440_MOESM1_ESM.docx]

Supplementary file 1: **Semi-structured key informant interview guide**

**Maternal and Perinatal Death Surveillance in Humanitarian Settings**

**Case-study Data Collection Tool**

**Interviewee:______________**

**Date:___________________**

**Introduction:** Thank you for agreeing to contribute to this effort to compile case studies of examples of maternal and perinatal death surveillance in humanitarian settings. This work is being done as a consultancy commissioned by WHO, and will inform discussions of an expert stakeholder meeting in October which will lead to recommendations for future practice and a research agenda.

| **Country example** *+* **Dates of data collection** |
| --- |
|  |
| **Type of crisis** *conflict/epidemic/natural disaster?* |
|  |
| **Phase of crisis** *acute/protracted?* |
|  |
| **Population included in data collection**  *(IDP/Refugee/Camp/Rural/Urban?)* |
|  |
| **Methodology** – *Prospective surveillance/retrospective survey? Review of records* *Community/facility based? How are community deaths identified + reviewed?* |
|  |
| **What guidance/documents were used to design the activity?** |
|  |
| **Linkage to other activities and health management information systems/civil registration and vital statistics, IDSR?** *E.g. nutrition screening, and other SRH indicators (Fertility rates, Facility delivery rates, contraception, sexual violence, antenatal attendance etc)* |
|  |
| **Link to National Ministry of Health MPSDR activities** *National committee ?* |
|  |
| **Legal framework –** *Protection of providers who report?* |
|  |
| **Denominator data – Data sources** *How collected ? Reliable? Pregnancies, total births, live births* |
|  |
| **Numerator data – Data sources** *Which deaths counted? Maternal/stillbirths/perinatal mortality/neonatal mortality? Pregnancy related, abortion related* |
|  |
| **Comparison of mortality data against other data collection methods/surveys?** |
|  |
| **Death reviews** – Who ? How often ? How done? **& Cause of death data** *How collected ? reliable? Used to change practice?* |
|  |
| **Cause of death – How determined? Coding? (ICD-10, ICD-MM, ICD-PM?)** |
|  |
| **Staff engagement & training–** *Who drives the process? Professional engagement? Acceptability/no blame culture? Supervision?* |
|  |
| **Resources / Cost effectiveness**  - *Time, costs?* *Who funds the activity?* |
|  |
| **Programmatic usage** - *How did the data inform the response?* |
|  |
| **Dissemination** - *What reports? Annual reports ? etc How shared? Which organisations? NGO/UN/National Gov ?* *With the community? Health cluster ?* |
|  |
| **Advocacy / media** – *Was the data used for advocacy? Did the data lead to increased resources? Was there media coverage, and was this useful?* *Any adverse outcomes?* |
|  |
| **Challenges / Bottlenecks** *– Data availability? Cost? Legal barriers? Acceptance?* |
|  |
| **What worked well?** *Has it been reproduced?* |
|  |
| **Lessons learned** |
|  |
| **Relevant documents to review** |
|  |
